# Predicting acute postsurgical pain in the postanesthesia care unit: risk tool development and internal validation

**DOI:** 10.1097/PR9.0000000000001329

**Published:** 2025-09-03

**Authors:** Nicholas Papadomanolakis-Pakis, Simon Haroutounian, Johan K. Sørensen, Charlotte Runge, Lone D. Brix, Christian F. Christiansen, Lone Nikolajsen

**Affiliations:** a Department of Clinical Medicine, Aarhus University, Aarhus, Denmark; b Department of Anaesthesiology and Intensive Care, Aarhus University Hospital, Aarhus, Denmark; c Department of Anesthesiology, Washington University School of Medicine in St. Louis, St. Louis, MO, USA; d Center for Elective Surgery, Silkeborg Regional Hospital, Silkeborg, Denmark; e Department of Anaesthesia and Intensive Care, Horsens Regional Hospital, Horsens, Denmark; f Department of Clinical Epidemiology, Aarhus University Hospital, Aarhus, Denmark

**Keywords:** Acute postoperative pain, Prediction, Prediction model development, Risk tool, Prognosis, Risk stratification

## Abstract

**Introduction:**

Moderate-to-severe acute postsurgical pain (APSP) is experienced by approximately 30% of surgical patients.

**Objectives:**

To improve early APSP management, we developed 2 pragmatic and generalizable point-of-care risk tools to preoperatively predict moderate-to-severe and severe APSP in the postanaesthesia care unit (PACU).

**Methods:**

This was a multicenter prospective cohort study of adult patients undergoing elective surgical procedures between May 2021 and May 2023. Candidate predictors were preidentified. Logistic regression was used to develop the models. Internal validation was conducted with bootstrap resampling. Model performance was assessed by discrimination and calibration. Decision curve analysis was conducted to evaluate clinical utility of the models.

**Results:**

The final cohort included 1380 patients; 45.1% experienced moderate-to-severe APSP and 12.4% experienced severe APSP in the PACU. Predictors retained in the final models independently associated with increased risk of both moderate-to-severe and severe pain included younger age, female sex, preoperative pain in the surgical area, preoperative opioid use, and longer expected surgery duration. Orthopedic surgery and regional anesthesia were associated with decreased risk of both outcomes. In addition, minimally invasive surgery was associated with increased risk of moderate-to-severe APSP, and other preoperative pain was associated with increased risk of severe APSP. Optimism-corrected area under the receiver operating characteristic curves were 0.75 and 0.72 for moderate-to-severe and severe APSP models, respectively. Both models demonstrated good calibration and beneficial clinical utility.

**Conclusion:**

Our models developed using point-of-care data on a heterogeneous surgery sample demonstrated acceptable performance and clinical utility for early APSP prediction. External validation is needed before implementation.

## Abstract 2

Supplemental Digital Content is Available in the Text.

Two risk tools were developed to preoperatively predict moderate-to-severe and severe acute postsurgical pain in the postanesthesia care unit. Both prediction models demonstrated acceptable predictive performance and clinical utility.

## 1. Introduction

Postsurgical pain is among the most prevalent pain conditions affecting a substantial proportion of patients who undergo surgery.^10,49^ An estimated 30% of surgical patients will experience moderate-to-severe acute postsurgical pain (APSP) and 11% will experience severe APSP,^7,31^ although higher estimates have been reported.^4,12,20,43^ Early acute postsurgical pain poses a significant challenge and burden to the health care system as it is associated with both short- and long-term negative outcomes including adverse physiological effects, prolonged postanesthesia care unit (PACU) length of stay, development of chronic postsurgical pain, and increased health care costs, highlighting the importance of early intervention.^6,8,9,11,21,30,34^ Early identification of high-risk patient profiles could facilitate informed discussions with patients, prompt consideration of proactive perioperative strategies, and tailored multimodal anesthesia to prevent unfavorable patient outcomes and improve PACU discharge times.^9,14,21^

Several prediction models have been developed to predict the likelihood of moderate and severe APSP in various surgery populations.^35^ Commonly identified predictors in previous models include age, surgery type, sex or gender, anxiety or surgical fear, preoperative pain intensity, preoperative analgesic use, pain catastrophizing, and expected incision size.^35^ Risk factors such as smoking status, history of depressive symptoms, and history of sleeping difficulties have also been associated with APSP.^18,51^ In addition, the influence of genetic polymorphisms, proteomics, and other novel biomarkers may have a role in postoperative pain prediction and targeted pain therapy.^23,24,26,41^ Determining which combination of predictors can produce accurate and beneficial predictions for APSP is crucial to improving perioperative pain outcomes. Machine learning approaches for predictive modelling have gained increased attention, however, concerns with data quality, generalizability, interpretability and clinical utility, with only marginal improvements in predictive performance, have limited their application in pain research.^19,28,38^ The development of simple and generalizable risk tools that use data available at point of care could increase adoption by clinicians and contribute to improved perioperative pain management and patient outcomes.

Identification of high-risk patients for APSP outcomes in the early postoperative period could improve postoperative pain management at a time when patients are most vulnerable to experiencing immediate pain and discomfort.^29,36^ Therefore, the purpose of this study was to develop 2 independent clinically applicable point-of-care risk tools to identify patients at high risk of both moderate-to-severe and severe APSP in the PACU. Given the mechanisms and contributing factors for moderate-to-severe and severe APSP may not be identical, the development of 2 distinct prediction models in parallel ensures the most relevant predictors are captured for each specific outcome. This approach aligns with the principles of personalized medicine enabling clinicians to make informed decisions and select a pain management approach tailored to individual patient needs and preferences. Although moderate pain is widely recognized as a threshold to initiate analgesic interventions to prevent moderate pain from escalating,^13^ identification of high-risk patients for severe pain could signal the need for more intensive monitoring and comprehensive intervention approaches. Overall, this study aims to bridge the gap between risk prediction and clinical implementation, providing pragmatic risk tools to support individualized perioperative pain management strategies in real-world clinical settings.

## 2. Methods

This study was granted ethics approval by the Regional Research Ethics Committee in Central Denmark Region (1-16-02-172-21; May 3, 2021). A study protocol outlining the statistical analysis plan was published^33^ and preregistered on ClinicalTrials.gov (NCT04866147, April 29, 2021) before patient enrollment. Written informed consent was obtained from all subjects and the study was conducted in accordance with the Danish Data Protection Act and Declaration of Helsinki. This study adhered to the Transparent Reporting of Multivariable Prediction Models for Individual Prognosis or Diagnosis (TRIPOD) guideline.^32^

### 2.1. Setting and data sources

This was a prospective multicenter cohort study conducted between May 2021 and May 2023. Patients were recruited from 3 hospitals in Denmark: Aarhus University Hospital (AUH), Silkeborg Regional Hospital (SRH), and Horsens Regional Hospital (HRH). Elective surgery patients at AUH were invited to participate and complete a preoperative questionnaire either by secure electronic mail within 5 days of their scheduled surgery or in person on the day of surgery. Patients at SRH were invited in person and given an envelope with the preoperative questionnaire during a routine preoperative appointment and asked to complete the questionnaire 1 day before surgery and return the envelope with the completed questionnaire on the day of surgery. Patients at HRH were invited in person and asked to complete the questionnaire on the day of surgery. Routinely collected patient information and clinical information were extracted from the electronic medical record.

### 2.2. Patient population

Consenting adult patients age 18 years and older undergoing elective surgical procedures under any type of anesthesia were included. Exclusion criteria included unwillingness to consent to participate, severe psychiatric illness, admission to ICU after surgery, and inadequate knowledge of the Danish language. A list of included surgical procedures can be found in Supplementary Table 1, http://links.lww.com/PR9/A339.

### 2.3. Model end points

The outcomes of interest were moderate-to-severe APSP and severe APSP measured on a 11-point numeric rating scale (NRS 0–10) where 0 = no pain and 10 = worst pain imaginable. To focus on the early postoperative period, acute postsurgical pain intensity scores were collected from patients on arrival to the postanesthesia care unit (PACU) and every 30 minutes for 3 hours or until discharge by nurses not involved in the study. Moderate-to-severe APSP was defined as maximum NRS pain intensity at rest ≥4^13^ and severe APSP defined as NRS ≥ 7^5^ in the first 3 postoperative hours. Pain intensity was rated as 0 if the patient was asleep. Outcome threshold cutoffs were defined according to the literature, with NRS ≥4 as an accepted threshold for initiating analgesic intervention for moderate pain and NRS ≥7 as a highly specific threshold for pain-related functional interference.^13^

### 2.4. Candidate predictors

We identified and defined 11 candidate predictors for inclusion in the multivariable models based on reviews,^42,51^ existing models, and clinical knowledge. All candidate predictors were required to be available at point of care—meaning at the time of preoperative consultation—and included age, sex, body mass index, smoking status (never smoked, former/current smoker), average preoperative pain intensity in the surgical area at rest in the past 1 week (NRS 0–10), presence of other preoperative pain (yes, no), preoperative opioid use in the past 1 week (yes, no), orthopedic surgery (yes, no), actual regional anesthesia (neuraxial, peripheral, none), expected surgical technique (open, minimally invasive), and expected surgery duration.

### 2.5. Sample size calculation

Sample size calculations for prediction models with binary outcomes were conducted for both APSP outcomes according to Riley et al.^37^ The sample size for moderate-to-severe APSP was based on a previous study by Tighe et al.,^47^ with an estimated Cox–Snell R^2^ value of 0.121, prevalence rate of 53%, and 12 degrees of freedom. Given these parameters, a minimum sample size of 835 patients and 36.9 events per variable were required to ensure a global shrinkage factor of ≥0.9, a small absolute difference of ≤0.05 in the apparent and adjusted Nagelkerke R^2^ value and precise estimate of overall risk within a margin of error of 0.05. Based on these assumptions, we expected 443 patients to experience moderate-to-severe APSP within our study sample.

The sample size for severe APSP was based on a study by Schnabel et al.^39^ with an estimated Cox–Snell R^2^ value of 0.966, prevalence rate of 25%, and 12 degrees of freedom. With these parameters, a minimum sample size of 1057 patients and 22 events per variable was required, and we expected 265 patients to experience severe APSP.

This cohort was also used to develop a prediction model for chronic postsurgical pain requiring a larger sample size than estimated for this study.^33^

### 2.6. Statistical analysis

Means, standard deviations (SDs), medians, and proportions were used to describe the study sample. A complete-case analysis was conducted since missing data accounted for <3% of the total sample and no single candidate predictor had missing values >5%. Missing data patterns were investigated using margin plots and distributions of missing variables and provided support that data were missing completely at random.^15^ A univariable analysis of the candidate predictors was conducted to assess the relationship between independent predictor variables and the outcomes. The linearity assumption for continuous variables was evaluated using restricted cubic splines by plotting continuous variables against the logit of the outcome. Restricted cubic splines were added to variables with evidence of nonlinearity.^15^ Collinearity of candidate predictors was assessed using variance inflation factors (<3 indicates low correlation).

Logistic regression was used to conduct multivariable analyses. All candidate predictors were entered into the models and a backwards stepwise selection approach was used to select the most predictive factors while maximizing Akaike's information criterion.^1^ Optimism was assessed through internal validation by bootstrapping with 500 repetitions to ascertain the best-fitted and most stable models.^45^ Model overfitting was corrected by shrinkage of predictor weights with the estimated slope value as the shrinkage factor multiplied with the pooled coefficients and a new intercept was estimated to align with the shrunken coefficients.^45^

Predictive model performance was evaluated by discrimination and calibration. For each model, the area under the receiver operating characteristic curve (AUROC) was assessed to determine how accurately predictions discriminate between individuals with and without the outcome. A model with an AUROC of 0.5 is no better than chance, whereas 1.0 represents perfect performance. In general, values ≥0.7 are considered “acceptable,” ≥0.8 “good,” and ≥0.9 “excellent.”^16^ Calibration plots, calibration slopes *b*, calibration intercepts, and Brier scores were assessed to evaluate the agreement between observed and predicted outcomes. A model is considered well calibrated when the calibration slope *b* is close to 1 and the calibration intercept is close to 0.^44^ Brier scores range between 0 and 1, where 0 indicates perfect accuracy and 1 indicates perfect inaccuracy. Overall model performance was assessed using Nagelkerke's pseudo-R^2^ and the le Cessie–van Houwelingen–Copas Hosmer unweighted sum of squares goodness-of-fit test.

A decision curve analysis was conducted to determine whether decisions made using the risk prediction models could be clinically beneficial compared with default “treat all” and “treat none” approaches and to quantify the net benefit of each model.^46^ The “treat all” approach can be considered a strategy that recommends additional treatment for all patients, whereas “treat none” denotes the control or reference treatment (ie, standard of care). To aid clinical interpretation of individual risks, threshold probability cutoffs were determined to classify patients as “low,” “moderate,” and “high risk.” The decision for threshold cutoffs were based on the treatment objectives of each model and corresponding levels of sensitivity and specificity. Model classification measures including sensitivity and specificity were also assessed. All analyses were conducted using R software V.4.3.1 (R Foundation for Statistical Computing, Vienna, Austria).

## 3. Results

### 3.1. Baseline patient characteristics and outcomes

**Figure 1.**
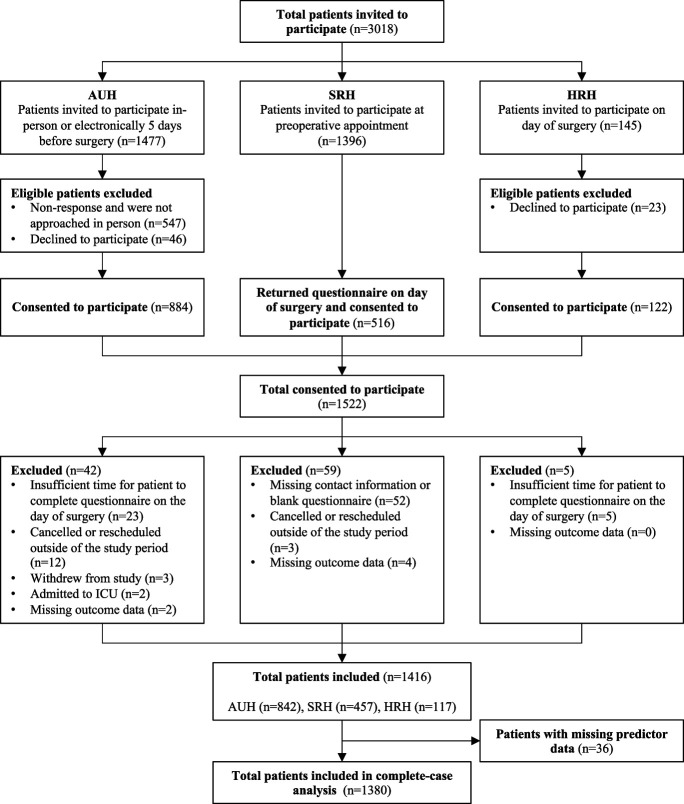
Patient recruitment flow diagram. AUH, Aarhus University Hospital; HRH, Horsens Regional Hospital; ICU, intensive care unit*;* SRH, Silkeborg Regional Hospital.

A total of 1522 patients consented to participate in the study (Fig. 1). After exclusions and loss to follow-up, 1416 (93%) patients were included. Of these, 1380 (97%) patients with complete data were analyzed. Patient and perioperative clinical characteristics stratified by recruitment site and percentage of missing data are summarized in Table 1. Incidence rates of moderate-to-severe APSP and severe APSP were 45.1% and 12.4%, respectively. See Supplementary Table 2, http://links.lww.com/PR9/A339, for perioperative characteristics stratified by surgery group.

**Table 1:** Baseline and perioperative characteristics.

	AUH (n = 842)	SRH (n = 457)	HRH (n = 117)	Overall (n = 1416)
Age (y), median [IQR]	55.0 [37.0, 67.0]	63.0 [51.0, 72.0]	56.0 [43.0, 73.0]	58.0 [44.0, 70.0]
Sex: female, n (%)	511 (60.7%)	237 (51.9%)	73 (62.4%)	821 (58.0%)
BMI, median [IQR]	25.9 [23.0, 29.4]	27.5 [24.3, 31.0]	26.2 [23.5, 29.4]	26.2 [23.5, 29.9]
Missing	2 (0.2%)	0 (0.0%)	0 (0.0%)	2 (0.1%)
Smoking status: former/current, n (%)	430 (51.1%)	249 (54.5%)	68 (58.1%)	747 (52.8%)
Missing	1 (0.1%)	4 (0.9%)	4 (3.4%)	9 (0.6%)
Preoperative pain in the surgical area, n (%)	489 (58.1%)	436 (95.4%)	78 (66.7%)	1003 (70.8%)
Preoperative pain at rest (NRS), mean (SD)	3.8 (2.3)	4.4 (2.4)	3.3 (2.1)	4.0 (2.4)
Preoperative pain on movement (NRS), mean (SD)	5.4 (2.5)	6.4 (2.1)	4.1 (2.5)	5.7 (2.4)
Pain medication use in the past 1 wk, n (%)*	265 (54.2%)	343 (75.1%)	50 (42.7%)	658 (46.5%)
Preoperative opioid use	37 (14.0%)	83 (24.2%)	15 (30.0%)	135 (20.5%)
Other preoperative pain, n (%)	356 (42.3%)	269 (58.9%)	49 (41.9%)	674 (47.6%)
Missing	10 (1.2%)	13 (2.8%)	5 (4.3%)	28 (2.0%)
Major surgery group, n (%)				
Orthopedic	423 (50.2%)	457 (100%)	0 (0.0%)	880 (62.1%)
Breast	193 (22.9%)	0 (0.0%)	0 (0.0%)	193 (13.6%)
Cardiothoracic	160 (19.0%)	0 (0.0%)	0 (0.0%)	160 (11.3%)
Abdominal	50 (5.9%)	0 (0.0%)	77 (65.8%)	127 (9.0%)
Genitourinary	15 (1.8%)	0 (0.0%)	40 (34.2%)	55 (3.9%)
Other	1 (0.1%)	0 (0.0%)	0 (0.0%)	1 (0.1%)
Surgical setting: Outpatient, n (%)	514 (61.0%)	274 (60.0%)	6 (5.1%)	794 (56.1%)
Expected surgical technique: Open, n (%)	468 (55.6%)	352 (77.0%)	6 (5.1%)	817 (57.7%)
Expected surgery duration (min), median [IQR]	60 [60, 116]	45.0 [45, 90]	90 [90, 120]	60 [45, 90]
Regional anesthesia, n (%)				
Peripheral block	459 (54.5%)	80 (17.5%)	1 (0.9%)	540 (38.1%)
Neuraxial block	108 (12.8%)	168 (36.8%)	1 (0.9%)	277 (19.6%)
PACU opioid dose (OME; first 3 h), mean (SD)	12.2 (20.7)	13.7 (18.4)	14.2 (21.4)	14.2 (21.4)
Acute postoperative pain, n (%)				
Moderate-to-severe (NRS ≥ 4)	394 (46.8%)	165 (36.1%)	80 (68.4%)	639 (45.1%)
Severe (NRS ≥ 7)	96 (11.4%)	50 (10.9%)	30 (25.6%)	176 (12.4%)

* Denominator is the proportion of patients who reported preoperative pain.

AUH, Aarhus University Hospital; BMI, body mass index; HRH, Horsens Regional Hospital; IQR, interquartile range; NRS, numeric rating scale; OME, oral morphine equivalents; PACU, postanesthesia care unit; SD, standard deviation; SRH, Silkeborg Regional Hospital.

### 3.2. Univariable analysis

Univariable associations between candidate predictors and the outcomes are presented in Table 2. Younger age, female sex, preoperative opioid consumption, and expected surgery duration were associated with higher risk of both moderate-to-severe APSP and severe APSP, while open surgical technique, orthopedic surgery, and regional anesthesia were associated with lower risk. In addition, former/current smoking status and presence of other preoperative pain were associated with higher risk of severe APSP.

**Table 2:** Univariable analysis for acute postsurgical pain outcomes.

	Moderate-to-severe APSP			Severe APSP		
Candidate predictors	OR	95% CI	*P*	OR	95% CI	*P*
Age (per 10 y)	0.89	0.84–0.95	0.000	0.86	0.78–0.94	0.000
Sex, female	1.53	1.23–1.90	0.000	1.69	1.20–2.39	0.003
BMI (per unit)	1.01	0.99–1.03	0.506	1.02	0.99–1.05	0.210
Smoking status, former/current	1.22	0.98–1.50	0.072	1.42	1.02–1.97	0.038
Preoperative pain (operative area) at rest (NRS)	1.03	0.99–1.07	0.192	1.10	1.04–1.17	0.000
Other preoperative pain	1.17	0.95–1.45	0.146	1.75	1.26–2.43	0.000
Preoperative opioid	1.90	1.33–2.75	0.000	2.95	1.91–4.47	0.000
Orthopedic surgery	0.42	0.34–0.52	0.000	0.62	0.45–0.86	0.004
Expected surgical technique, open	0.55	0.44–0.68	0.000	0.69	0.50–0.96	0.025
Expected surgery duration (per 30 min)	1.55	1.42–1.69	0.000	1.41	1.28–1.56	0.000
Regional anesthesia						
Neuraxial block	0.16	0.11–0.22	0.000	0.21	0.10–0.37	0.000
Peripheral block	0.58	0.46–0.73	0.000	0.58	0.41–0.81	0.002

CI, confidence interval; OR, odds ratio.

### 3.3. Model development and performance

#### 3.3.1. Moderate-to-severe acute postsurgical pain

**Figure 2.**
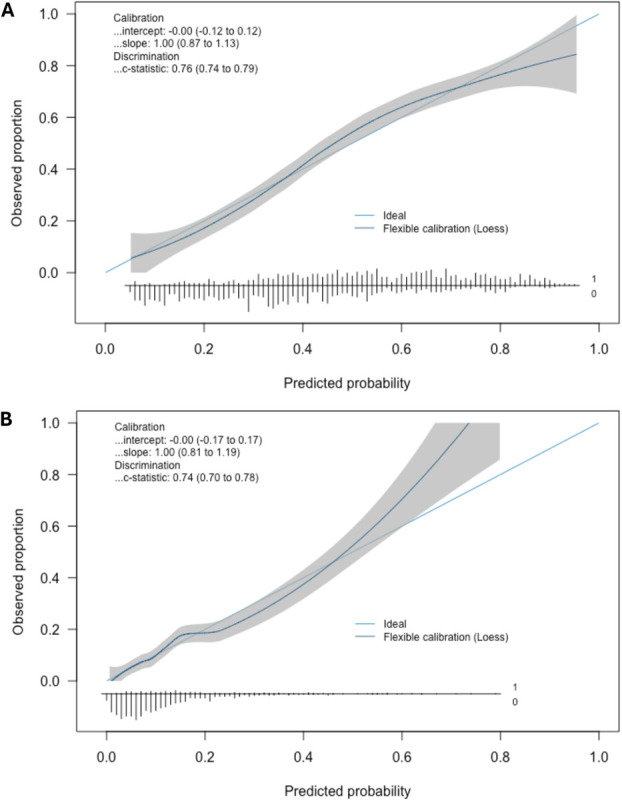
Calibration plots. (A) Calibration of the model predicting moderate-to-severe APSP shows risks may be overestimated at the extremes of ranges. (B) Calibration of the model predicting severe APSP shows underestimation of risks for patients with higher predicted risks. Ticks on the *x*-axis represent the frequency distribution of the prediction probabilities (1 = event, 0 = nonevent). Shaded area, 95% confidence interval along the calibration curve.

The final prediction model for moderate-to-severe APSP after predictor selection included age, sex, preoperative pain in the surgical area at rest, preoperative opioid consumption, orthopedic surgery, expected surgical technique, expected surgery duration, and regional anesthesia (Table 3). The AUROC of the reduced model predicting moderate-to-severe APSP was 0.762 (95% CI 0.737–0.787), optimism-corrected to 0.754 (Table 4; Supplementary Figure 1A, http://links.lww.com/PR9/A339). The optimism-corrected calibration slope was 0.96 and the calibration intercept was −0.006 (Table 4). Inspection of the calibration plot for the reduced model suggests the model is well calibrated but shows risks may be slightly overestimated at higher probabilities (Fig. 2A). Calibration plots stratified by sex and surgery group are presented in Supplementary Figure 2, http://links.lww.com/PR9/A339. Instability plots suggest predictions generated by the developed model are relatively stable, supporting the reliability of individual risk estimates (Supplementary Figure 4, http://links.lww.com/PR9/A339).

**Table 3:** Unadjusted and penalized model coefficients.

Candidate predictors	Moderate-to-severe postoperative pain				Severe postoperative pain			
	Full model		Reduced model		Full model		Reduced model	
	Estimate (SE)	Penalized estimate (SE)	Estimate (SE)	Penalized estimate (SE)	Estimate (SE)	Penalized estimate (SE)	Estimate (SE)	Penalized estimate (SE)
Intercept	−0.056 (0.834)	−0.061 (0.834)	−0.613 (0.418)	−0.597 (0.418)	−2.360 (0.787)	−2.288 (0.787)	−1.888 (0.700)	−1.871 (0.700)
Age + spline (model 2)	−0.013 (0.004)	−0.012 (0.004)	−0.012 (0.004)	−0.012 (0.004)	−0.017 (0.013) −0.004 (0.013)	−0.015 (0.012) −0.004 (0.013)	−0.014 (0.012) −0.006 (0.013)	−0.013 (0.012) −0.006 (0.013)
Sex, female	0.370 (0.131)	0.352 (0.131)	0.389 (0.128)	0.375 (0.128)	0.478 (0.192)	0.426 (0.192)	0.426 (0.191)	0.388 (0.191)
BMI + spline (model 1)	−0.029 (0.032) 0.059 (0.045)	−0.027 (0.032) 0.056 (0.045)	—	—	0.015 (0.017)	0.013 (0.017)	—	—
Smoking status, former/current	0.123 (0.127)	0.117 (0.127)	—	—	0.325 (0.186)	0.290 (0.186)	—	—
Preoperative pain in the operative area at rest (NRS)	0.110 (0.029)	0.105 (0.029)	0.120 (0.029)	0.116 (0.029)	0.122 (0.040)	0.108 (0.040)	0.129 (0.039)	0.117 (0.039)
Other pain	0.196 (0.128)	0.186 (0.128)	—	—	0.549 (0.185)	0.490 (0.185)	0.538 (0.184)	0.489 (0.184)
Preoperative opioid	0.564 (0.230)	0.537 (0.229)	0.607 (0.227)	0.585 (0.227)	0.713 (0.263)	0.636 (0.263)	0.775 (0.258)	0.705 (0.258)
Orthopedic surgery	−0.692 (0.169)	−0.658 (0.169)	−0.701 (0.167)	−0.675 (0.167)	−0.585 (0.235)	−0.522 (0.235)	−0.642 (0.230)	−0.584 (0.230)
Regional block								
None (referent)	—	—	—	—	—	—	—	—
Peripheral	−0.691 (0.148)	−0.657 (0.148)	−0.704 (0.147)	−0.678 (0.147)	−0.261 (0.206)	−0.233 (0.206)	−0.240 (0.205)	−0.218 (0.205)
Neuraxial	−1.362 (0.210)	−1.295 (0.210)	−1.347 (0.209)	−1.298 (0.209)	−0.930 (0.360)	−0.830 (0.360)	−1.009 (0.354)	−0.918 (0.354)
Expected surgical technique, open	−0.351 (0.138)	−0.471 (0.132)	−0.338 (0.137)	−0.325 (0.137)	−0.259 (0.196)	−0.231 (0.196)	—	—
Expected surgery duration (min) + spline (model 1 and model 2)	0.022 (0.008) 0.006 (0.049) −0.057 (0.115)	0.008 (0.008) 0.071 (0.048) −0.190 (0.112)	0.022 (0.008) 0.008 (0.049) −0.064 (0.114)	0.021 (0.008) 0.008 (0.049) −0.061 (0.114)	−0.001 (0.013) 0.067 (0.072) −0.147 (0.164)	−0.001 (0.012) 0.059 (0.072) −0.131 (0.164)	−0.004 (0.012) 0.089 (0.070) −0.202 (0.161)	−0.004 (0.012) 0.081 (0.070) −0.184 (0.161)

SE, standard error.

**Table 4:** Model performance measures.

Prediction model	R^2^	Optimism-corrected R^2^	AUC (95% CI)	Optimism-corrected AUC	Calibration slope *b*	Calibration intercept	Brier score	CHCH test *P*
Moderate-to-severe APSP								
Full model	0.27	0.25	0.764 (0.739–0.789)	0.753	0.95	−0.006	0.20	0.58
Reduced model	0.26	0.25	0.762 (0.737–0.787)	0.754	0.96	−0.006	0.20	0.51
Severe APSP								
Full model	0.18	0.15	0.748 (0.709–0.787)	0.724	0.89	−0.18	0.10	0.57
Reduced model	0.18	0.15	0.743 (0.705–0.782)	0.724	0.91	−0.15	0.10	0.68

AUROC, area under the receiver operating curve; CHCH, le Cessie–van Houwelingen–Copas Hosmer; CI, confidence interval; R^2^, Nagelkerke's pseudo-R^2^.

Decision curve analysis demonstrated superior net benefit (NB) of the model across a wide range of threshold probabilities compared to default “treat all” and “treat none” strategies (Supplementary Figure 5, http://links.lww.com/PR9/A339). Considering treatment goals for moderate-to-severe APSP, along with the risks and benefits of classifying patients at different risk thresholds and corresponding sensitivity and specificity, suggested probability thresholds were determined. A threshold of >50% can be considered “high-risk,” 40% to 50% as “moderate-risk,” and <40% as “low-risk.” The high-risk threshold probability corresponds to a sensitivity of 0.61 and a specificity of 0.76.

#### 3.3.2. Severe acute postsurgical pain

The final model predicting severe APSP included age, sex, preoperative pain in the surgical area at rest, other preoperative pain, preoperative opioid consumption, orthopedic surgery, expected surgery duration, and regional anesthesia (Table 3). The AUROC of the reduced model predicting severe APSP was 0.743 (95% CI 0.705–0.782), optimism-corrected to 0.724 (Table 4; Supplementary Figure 1B, http://links.lww.com/PR9/A339). The optimism-corrected calibration slope was 0.91 and calibration intercept was −0.15. Inspection of the calibration plot for severe APSP suggests risks may be slightly underestimated at higher probabilities (Fig. 2B). Stratified calibration plots by sex and surgery group for the model predicting severe APSP are presented in Supplementary Figure 3, http://links.lww.com/PR9/A339. Instability plots are presented in Supplementary Figure 4, http://links.lww.com/PR9/A339.

Decision curve analysis for severe APSP also demonstrated superior NB across a wide range of threshold probabilities compared with “treat all” and “treat none” strategies (Supplementary Figure 5, http://links.lww.com/PR9/A339). Considering treatment goals for severe APSP, the following probability thresholds are suggested: >20% as “high-risk”, 10% to 20% as “moderate-risk,” and <10% as “low-risk.” The high-risk threshold probability corresponds to a sensitivity 0.38 and a specificity of 0.86 (Supplementary Tables 3 and 4, http://links.lww.com/PR9/A339).

### 3.4. Individual risk prediction

Risk formulas and hypothetical patient examples to illustrate the intended clinical use of each model can be found in Appendix B, http://links.lww.com/PR9/A339, of the Supplement. A web-based risk calculator was developed and can be accessed at https://psp-risktools.shinyapps.io/psp_risk/.

## 4. Discussion

Two prediction models for moderate-to-severe APSP and severe APSP were developed and internally validated using clinically relevant predictors available at point of care to identify high-risk patients for inadequately controlled APSP in the PACU. Both models demonstrated acceptable discrimination and good calibration.

Various prediction models have been developed to estimate APSP risk.^35^ The largest study, a retrospective cohort of 234,274 patients from 4 US hospitals, used electronic health record data to predict APSP on postoperative days (POD) 0 to 4.^27^ This model incorporated 5011 predictors achieving its highest predictive performance for moderate-to-severe APSP on POD 1 (AUROC = 0.79). Another study based on the PAIN OUT registry including 50,005 patents from 23 European countries used 4 predictors to predict severe APSP on POD 1 with fair discrimination (AUROC = 0.605).^39^ Although these models benefit from large sample sizes, use of registry or retrospective data can introduce bias and limit applicability.^25,50^ In contrast to the aforementioned models that primarily focus on broader postoperative periods, our emphasis on predicting early APSP provides an opportunity to guide prompt and proactive interventions in the PACU. Our model also demonstrated improved performance compared with a randomized controlled trial (RCT)–based model of 1944 patients from the Netherlands predicting severe APSP in the first postoperative hour (AUROC = 0.75 vs 0.71).^20^ Although RCTs are valuable, they often include strict inclusion criteria limiting generalizability.^50^

Contrary to previous research indicating open surgery as a predictor for greater APSP severity,^17,40^ we found minimally invasive surgery to be associated with higher risk of moderate to severe APSP. A post hoc analysis suggested this may be influenced by regional anesthesia, as the protective effect of a peripheral nerve block was greater in open surgery than in minimally invasive surgery. Although the interaction effect was significant, it had minimal impact on model performance with adjusted AUROCs increasing slightly from 0.754 to 0.756 for moderate-to-severe and 0.724 to 0.732 for severe APSP models, with no impact on calibration. Given this interaction was not specified a priori, its inclusion could increase the risk of overfitting, given the size of the cohort and reduce generalizability. Since the overall predictive ability of each model remained relatively unaffected, the models can still be applied as intended. The observed findings could also reflect the heterogeneity of the surgical cohort, suggesting the effect of surgical technique might become more evident when comparing patients undergoing the same surgical procedure (ie, thoracotomy vs video-assisted thoracoscopic lobectomy).^3^ Other unmeasured confounders such as surgical complexity and patient comorbidities could have influenced these results, however data on these factors were not available for further exploration.

Our study found orthopedic surgery was associated with lower risk of APSP outcomes. Orthopedic procedures on the extremities are typically associated with higher postsurgical pain scores on the first postoperative day.^12^ In our cohort, abdominal surgeries had the highest incidence of moderate-to-severe APSP (72%) and the second highest incidence of severe APSP (21%). This aligns with a large prospective study measuring immediate postoperative pain in the operating room and PACU, where laparoscopic cholecystectomy and other abdominal surgeries were among the most painful procedures.^36^ A large retrospective study also found abdominal, thoracic, and urologic procedures were associated with increased risk of severe APSP on POD 1, compared with orthopedic, spinal, and head and neck procedures.^2^ The consistent finding of high APSP incidence rates after abdominal surgeries suggests there is a need for targeted interventions to adequately manage APSP in this patient population. The lower incidence of APSP among orthopedic procedures in our cohort may also reflect a higher proportion of patients in this subgroup who received successful regional or spinal anesthesia, although this cannot be confirmed by our data.

Inclusion of regional anesthesia as a preoperative predictor of APSP aligns with anesthesia practice where nerve block administration is typically planned before surgery. Omitting this information could bias the model by disregarding a significant determinant of APSP intensity.^9^ Previous predictive studies have not incorporated this factor, but understanding its effect on individual risk estimates could lead to more effective pain management. For high-risk patients where regional anesthesia is not initially planned, its consideration could be viable. Identification of high-risk patients despite planned regional anesthesia could signal the need for additional interventions to ensure adequate APSP management.

Reliable risk prediction tools can support clinicians in developing tailored treatment strategies to prevent and manage unfavorable APSP outcomes.^22^ We have demonstrated the ability to develop reasonable and generalizable models using point-of-care data to predict early APSP. Decision curve analysis suggests model-based decisions for both outcomes could benefit APSP management. Although risk probability cutoffs were suggested, classifying a patient's risk should take into account specific treatment factors in addition to predicted risk probabilities, as values associated with potential harms and benefits for particular interventions vary, as do clinician preferences.^48^ Clinicians more risk-averse to APSP may opt for a lower risk threshold, while clinicians risk-averse to a particular treatment may prefer a higher risk threshold. Ultimately, classification of “high-risk” patients should involve shared decision-making.^22^

Our study has several strengths. The prospective cohort was created specifically for prediction model development and the multicenter design provides a greater degree of generalizability of predictions. Sample size calculations were based on studies with a diverse range of surgical procedures ensuring applicability to our study design and performed independent of events-per-variable.^37^ Restricted cubic splines were used to model nonlinear relationships between continuous predictors and associated outcomes. Decision curve analysis demonstrated beneficial clinical utility of our model in comparison with default treatment strategies.

This study also has limitations. First, the cohort was heterogeneous with a wide variety of inpatient and outpatient procedures. This was deliberate for our objective to increase generalizability across a wider range of surgery populations.

Second, because of resource limitations, the cohort reflects a convenience sample that may have resulted in sampling bias. Attempts to mitigate this bias included sampling from different surgery types and settings.

Third, our sample size calculations were based on studies with higher APSP incidence rates than observed in our cohort, particularly for severe APSP (12.4% vs 25% in Schnabel et al.^39^), likely attributed to the timing of outcome measurement (3 vs 24 hours postoperatively). Although this may increase risk of overfitting, instability plots indicated reasonably stable models despite differences in incidence rates. Minor differences in patient demographics (ie, slightly higher mean age and proportion of females) may have also limited the applicability of previous incidence rate estimates to our study; however, we do not expect these to affect our findings.

Fourth, as our aim was to develop a risk stratification tool for use in clinical settings, only point-of-care predictors were included. Although psychological and experimental factors have potential to improve predictive performance, it is important to consider that obtaining data on such predictors is relatively time-intensive and often requires specialized materials or equipment.

Fifth, surgical procedures were dichotomized (orthopedic vs other) to simplify the model, sacrificing specificity. Although this broad categorization may not capture differences in tissue trauma across procedure types, we adjusted for surgical technique to account for some variation in procedure severity.

Finally, since these models aim to improve early postsurgical pain management, APSP measurement was restricted to the PACU as the effects of anesthesia subside. Although this time frame does not capture longer-term pain trajectories, it provides an opportunity to improve early pain control, mobilization, and PACU length of stay.^6,21^ Complementary models could be developed to predict pain over longer postoperative periods to ensure a comprehensive pain management approach.

It could benefit clinicians if future studies evaluated the clinical effectiveness of risk stratification tools for APSP to determine their impact on patient outcomes in real-world clinical settings. Eventual ability to predict which patients respond favorably to specific treatments could allow a directive risk-based approach to present therapeutic options for APSP. Finally, implementation of our models as an artificial intelligence platform could facilitate self-learning capabilities through ongoing collection of predictor and outcome information and continuous evaluation and improvement of predictive accuracy over time.

## 5. Conclusions

Our models, developed using point-of-care data on a heterogeneous surgery cohort, demonstrated acceptable predictive performance and clinical utility for APSP outcomes in the PACU. After external validation, our models could be used to identify high-risk patients for targeted perioperative interventions and facilitate enrolment in randomized trials aimed at testing novel strategies to improve early APSP management.

## Disclosures

S.H. has received research support from Disarm Therapeutics and consultancy fees from Vertex Pharmaceuticals unrelated to this project. No other authors report conflicts of interest.

## Supplemental digital content

Supplemental digital content associated with this article can be found online at http://links.lww.com/PR9/A339.

## Supplementary Material
