# Supplementary material for: Predicting acute postsurgical pain in the postanesthesia care unit: risk tool development and internal validation
Source: Pain Rep. 2025 Sep 3;10(5):e1329. doi: 10.1097/PR9.0000000000001329 (PMC12410311; doi:10.1097/PR9.0000000000001329)
Supplement: Supplementary file 1 [file painreports-10-e1329-s001.pdf]

Supplementary Material

Appendix A

Table S1. Included surgical procedures and corresponding procedure codes.

Table S2. Patient and clinical characteristics stratified by surgery group.

Table S3. Net benefit at different risk thresholds.

Table S4. Prediction classification measures at different risk thresholds.

Figure S1. AUROC plots.

Figure S2. Calibration plots stratified by sex.

Figure S3. Calibration plots stratified by surgery group.

Figure S4. Instability plots for final APSP models.

Figure S5. Decision curves.

Appendix B

Risk formulas and hypothetical patient examples

Table S1. Included surgical procedures and corresponding 3- or 4-digit hospital procedure codes.

Surgery group	3-or 4-digit hospital procedure codes	Procedure description
Orthopedic	KAB<br>KAC<br>KNA<br>KNB<br>KNC<br>KND<br>KNE<br>KNF<br>KNG<br>KNH<br>KQA<br>KYN	Operations on spinal cord and nerve roots<br>Operations on peripheral nerves<br>Back and neck surgeries<br>Shoulder and upper arm surgeries<br>Elbow and forearm surgeries<br>Operations on wrist and hand<br>Pelvic surgeries<br>Hip and thigh surgeries<br>Knee and lower leg surgeries<br>Ankle and foot surgeries<br>Operations on skin and subcutaneous tissue of head and neck<br>Removal of grafts from the musculoskeletal system
Cardiothoracic	KFK<br>KFM<br>KFN<br>KGA<br>KGD<br>KGE<br>KPJ	Mitral valve surgery<br>Aortic valve surgery<br>Operations on coronary arteries<br>Operations on chest wall, pleura and diaphragm<br>Lung surgery<br>Operations on the mediastinum<br>Operations on the lymphatic system
Breast	KHAB<br>KHAC<br>KHAD<br>KHAE	Breast resections<br>Mastectomies<br>Corrective breast surgeries<br>Breast reconstructive surgeries
Abdominal	KJA<br>KJB<br>KJE<br>KJF<br>KJK	Operations on abdominal wall, peritoneum, mesentery and omentum<br>Diaphragmatic operations and operations on gastroesophageal reflux<br>Operations on the appendix<br>Small bowel and colon surgeries<br>Bile duct surgeries
Genitourinary	KKB<br>KKF<br>KKG<br>KLA<br>KLC<br>KLF	Urinary tract surgeries<br>Operations on the scrotum and scrotal contents<br>Penis surgeries<br>Ovarian surgery<br>Operations on uterus and parameters<br>Operations on the vulva and perineum
Other	KBA	Thyroid surgeries

Table S2. Patient and clinical characteristics stratified by surgery group.

	Cardiothoracic (N=160)	Breast (N=193)	Abdominal (N=127)	Genitourinary (N=55)	Orthopedic (N=880)	Other (N=1)	Overall (N=1416)
Age (yr), median [IQR]	68.0 [60.8, 74.0]	56.0 [47.0, 64.0]	59.0 [42.5, 71.0]	44.0 [39.0, 51.0]	56.0 [39.0, 69.0]	63.0 [63.0, 63.0]	58.0 [44.0, 70.0]
Sex: female, n (%)	66 (41.3%)	192 (99.5%)	71 (55.9%)	41 (74.5%)	450 (51.1%)	1 (100%)	821 (58.0%)
BMI, median [IQR]	26.4 [23.7, 30.8]	25.0 [22.4, 27.9]	26.6 [24.0, 30.3]	25.7 [23.3, 28.8]	26.6 [23.8, 30.1]	27.8 [27.8, 27.8]	26.2 [23.5, 29.9]
Missing	0 (0%)	0 (0%)	0 (0%)	0 (0%)	2 (0.2%)	0 (0%)	2 (0.1%)
Smoking status: Former/current, n (%)	127 (79.4%)	89 (46.1%)	68 (53.5%)	28 (50.9%)	434 (49.3%)	1 (100%)	747 (52.8%)
Missing	0 (0%)	0 (0%)	3 (2.4%)	1 (1.8%)	5 (0.6%)	0 (0%)	9 (0.6%)
Preoperative pain in the surgical area, n (%)	28 (17.5%)	39 (20.2%)	81 (63.8%)	33 (60.0%)	822 (93.4%)	0 (0%)	1003 (70.8%)
Preoperative pain at rest (NRS), mean (SD)	0.5 (1.33)	0.6 (1.35)	2.3 (2.56)	1.7 (2.04)	3.9 (2.53)	NA	2.8 (2.69)
Preoperative pain on movement (NRS), mean (SD)	0.5 (1.47)	0.6 (1.53)	2.6 (2.89)	1.8 (2.35)	5.7 (2.61)	NA	4.0 (3.30)
Pain medication use in the past 1 week, n (%)*	9 (32.1%)	8 (20.1%)	48 (59.3%)	20 (60.6%)	573 (69.7%)	0 (0%)	658 (65.6%)
Preoperative opioid use	2 (22.2%)	1 (12.5%)	13 (27.1%)	6 (30.0%)	113 (19.7%)	0 (0%)	135 (20.5%)
Other preoperative pain, n (%)	53 (33.1%)	76 (39.4%)	55 (43.3%)	21 (38.2%)	468 (53.2%)	1 (100%)	674 (47.6%)
Missing	2 (1.3%)	1 (0.5%)	7 (5.5%)	0 (0%)	18 (2.0%)	0 (0%)	28 (2.0%)
Surgical setting: Outpatient, n (%)	0 (0%)	158 (81.9%)	46 (36.2%)	16 (29.1%)	574 (65.2%)	0 (0%)	794 (56.1%)
Regional anesthesia, n (%)							
Peripheral block	91 (56.9%)	123 (63.7%)	21 (16.5%)	10 (18.2%)	295 (33.5%)	0 (0%)	540 (38.1%)
Neuraxial block	26 (16.3%)	0 (0%)	2 (1.6%)	0 (0%)	249 (28.3%)	0 (0%)	277 (19.6%)
Expected surgical technique: Open, n (%)	62 (38.8%)	192 (99.5%)	7 (5.5%)	14 (25.5%)	550 (62.5%)	1 (100%)	826 (58.3%)
Expected surgery duration, median [IQR]	120 [120, 159]	60.0 [60.0, 90.0]	90.0 [60.0, 120]	90.0 [60.0, 120]	60.0 [45.0, 75.0]	90.0 [90.0, 90.0]	60.0 [45.0, 90.0]
PACU opioid dose (OME; first 3 hours), median [IQR]	10.0 [0, 26.1]	0 [0, 19.5]	22.5 [8.25, 37.5]	20.0 [0, 42.0]	5.00 [0, 20.0]	15.0 [15.0, 15.0]	7.50 [0, 22.5]
Acute postoperative pain, n (%)							
Moderate-to-severe (NRS ≥4)	106 (66.3%)	83 (43.0%)	92 (72.4%)	31 (56.4%)	326 (37.0%)	1 (100%)	639 (45.1%)
Severe (NRS ≥7)	31 (19.4%)	14 (7.3%)	26 (20.5%)	14 (25.5%)	91 (10.3%)	0 (0%)	176 (12.4%)

**Denominator is the proportion of patients who reported preoperative pain.* *BMI* Body mass index; *IQR* Interquartile range; *NRS* Numeric rating scale; *OME* Oral morphine equivalents; *PACU* Post-anesthesia care unit; *PCS* Pain Catastrophizing Scale; *SD* Standard deviation.

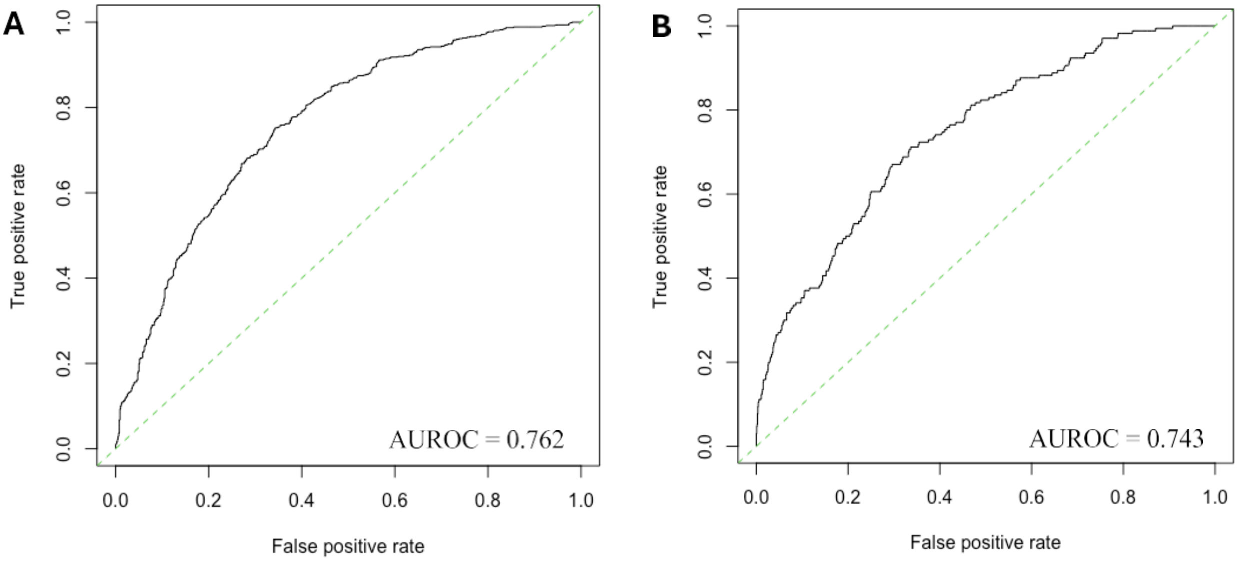

Figure S1. AUROC plots. (A) Model predicting moderate-to-severe APSP; (B) Model predicting severe APSP.

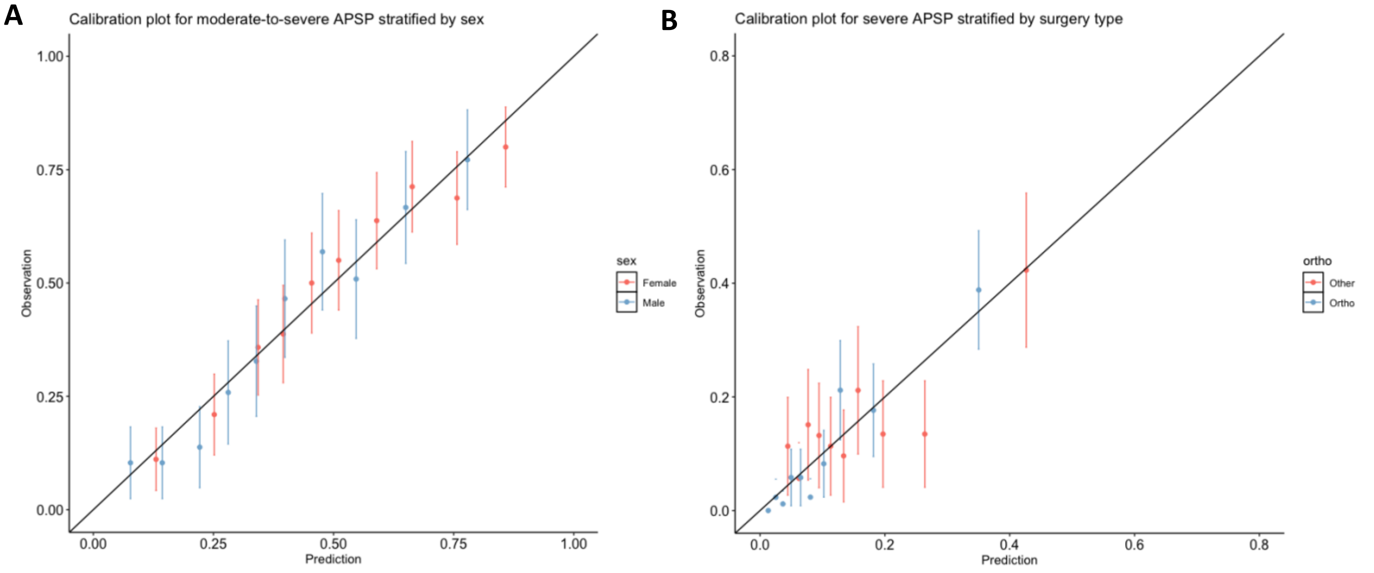

Figure S2. Calibration plots stratified by sex illustrating similar calibration between female and male sex. (A) Model predicting moderate-to-severe APSP; (B) Model predicting severe APSP.

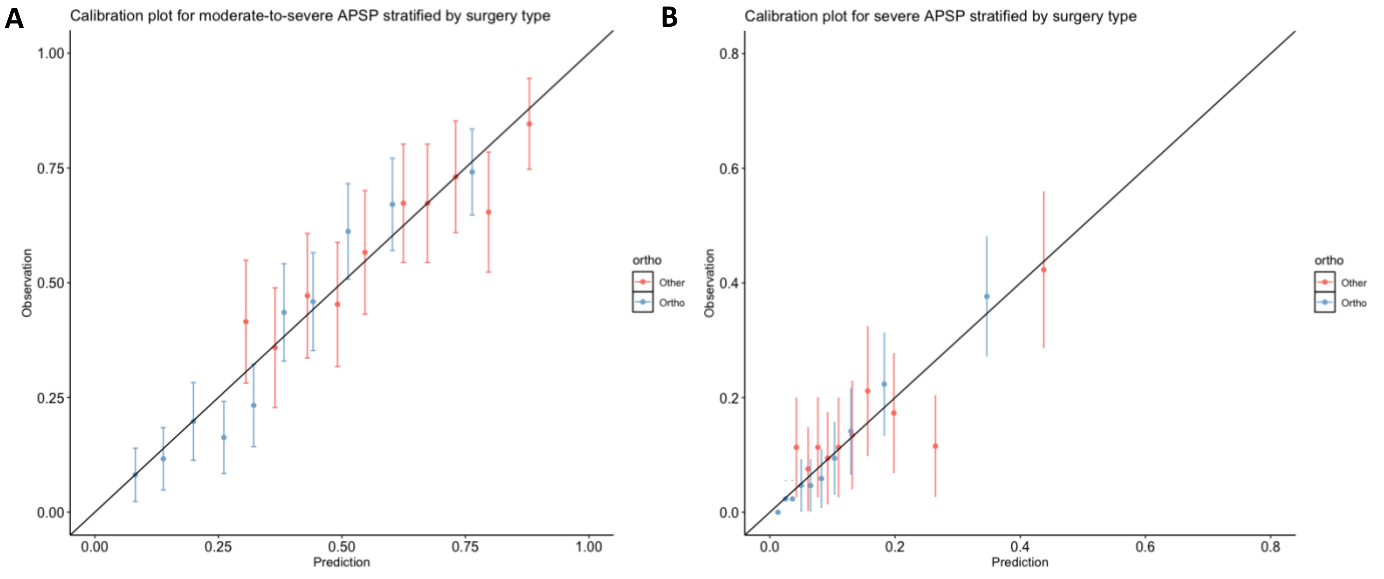

Figure S3. Calibration plots stratified by surgery group illustrating similar calibration between orthopedic surgery and all other surgery types. (A) Model predicting moderate-to-severe APSP; (B) Model predicting severe APSP.

Table S3. Net benefit at different risk thresholds.

**Moderate-to-severe APSP**			**Severe APSP**		
**Strategy**	**Decision threshold**	**Net benefit**	**Strategy**	**Decision threshold**	**Net benefit**
Treat all	10%	0.390	Treat all	5%	0.077
Treat all	20%	0.313	Treat all	10%	0.026
Treat all	30%	0.215	Treat all	15%	-0.032
Treat all	40%	0.085	Treat all	20%	-0.096
Treat all	50%	-0.099	Treat all	25%	-0.169
Treat all	60%	-0.373	Treat all	30%	-0.253
Treat none	10%	0.000	Treat none	5%	0.000
Treat none	20%	0.000	Treat none	10%	0.000
Treat none	30%	0.000	Treat none	15%	0.000
Treat none	40%	0.000	Treat none	20%	0.000
Treat none	50%	0.000	Treat none	25%	0.000
Treat none	60%	0.000	Treat none	30%	0.000
Prediction model	10%	0.390	Prediction model	5%	0.082
Prediction model	20%	0.331	Prediction model	10%	0.053
Prediction model	30%	0.276	Prediction model	15%	0.031
Prediction model	40%	0.210	Prediction model	20%	0.016
Prediction model	50%	0.142	Prediction model	25%	0.017
Prediction model	60%	0.089	Prediction model	30%	0.015

Table S4. Prediction classification measures at different risk thresholds.

Risk threshold	Sensitivity (95% CI)	Specificity (95% CI)	PPV (95% CI)	NPV (95% CI)
**Moderate-to-severe APSP (NRS ≥4)**				
10%	0.99 (0.98–1.00)	0.09 (0.07–0.10)	0.47 (0.45–0.49)	0.90 (0.84–0.95)
20%	0.96 (0.95–0.97)	0.25 (0.23–0.28)	0.51 (0.49–0.54)	0.89 (0.85–0.93)
30%	0.91 (0.89–0.93)	0.43 (0.40–0.45)	0.57 (0.54–0.59)	0.85 (0.82–0.88)
40%	0.77 (0.74–0.80)	0.62 (0.59–0.65)	0.63 (0.60–0.65)	0.77 (0.74–0.80)
42% (Youden cut-point)	0.75 (0.67–0.83)	0.66 (0.58–0.74)	0.64 (0.61–0.69)	0.76 (0.70–0.80)
45% (Prevalence/index of union)	0.69 (0.66–0.72)	0.70 (0.67–0.72)	0.65 (0.62–0.68)	0.74 (0.70–0.77)
47% (Minimize misclassification)	0.67 (0.60–0.78)	0.73 (0.64–0.79)	0.67 (0.63–0.71)	0.73 (0.70–0.78)
50%	0.61 (0.58–0.64)	0.76 (0.74–0.78)	0.68 (0.64–0.71)	0.70 (0.68–0.73)
60%	0.45 (0.42–0.48)	0.86 (0.84–0.88)	0.73 (0.69–0.77)	0.66 (0.63–0.68)
70%	0.26 (0.23–0.29)	0.93 (0.91–0.94)	0.75 (0.70–0.79)	0.61 (0.58–0.63)
**Severe APSP (NRS ≥7)**				
5%	0.94 (0.90–0.96)	0.27 (0.25–0.29)	0.15 (0.13–0.17)	0.97 (0.95–0.98)
10%	0.75 (0.69–0.80)	0.59 (0.56–0.61)	0.20 (0.18–0.23)	0.94 (0.93–0.96)
11% (Youden cut-point)	0.71 (0.63–0.81)	0.66 (0.55–0.75)	0.23 (0.20–0.27)	0.94 (0.93–0.96)
12% (Prevalence/index of union)	0.67 (0.61–0.73)	0.69 (0.67–0.72)	0.24 (0.20–0.27)	0.94 (0.92–0.95)
15%	0.54 (0.47–0.59)	0.78 (0.76–0.80)	0.25 (0.22–0.29)	0.92 (0.91–0.94)
20%	0.38 (0.32–0.45)	0.86 (0.84–0.87)	0.28 (0.22–0.33)	0.91 (0.89–0.92)
25%	0.34 (0.28–0.40)	0.92 (0.90–0.93)	0.36 (0.30–0.43)	0.91 (0.89–0.92)
30%	0.26 (0.21–0.32)	0.95 (0.94–0.96)	0.45 (0.37–0.52)	0.90 (0.89–0.92)
35%	0.20 (0.15–0.26)	0.97 (0.97–98)	0.52 (0.41–0.61)	0.90 (0.88–0.91)
52% (Minimize misclassification)	0.11 (0.06–0.20)	0.996 (0.99–1.00)	0.78 (0.61–0.94)	0.89 (0.88–0.90)

PPV Positive predictive value; *NPV* Negative predictive value.

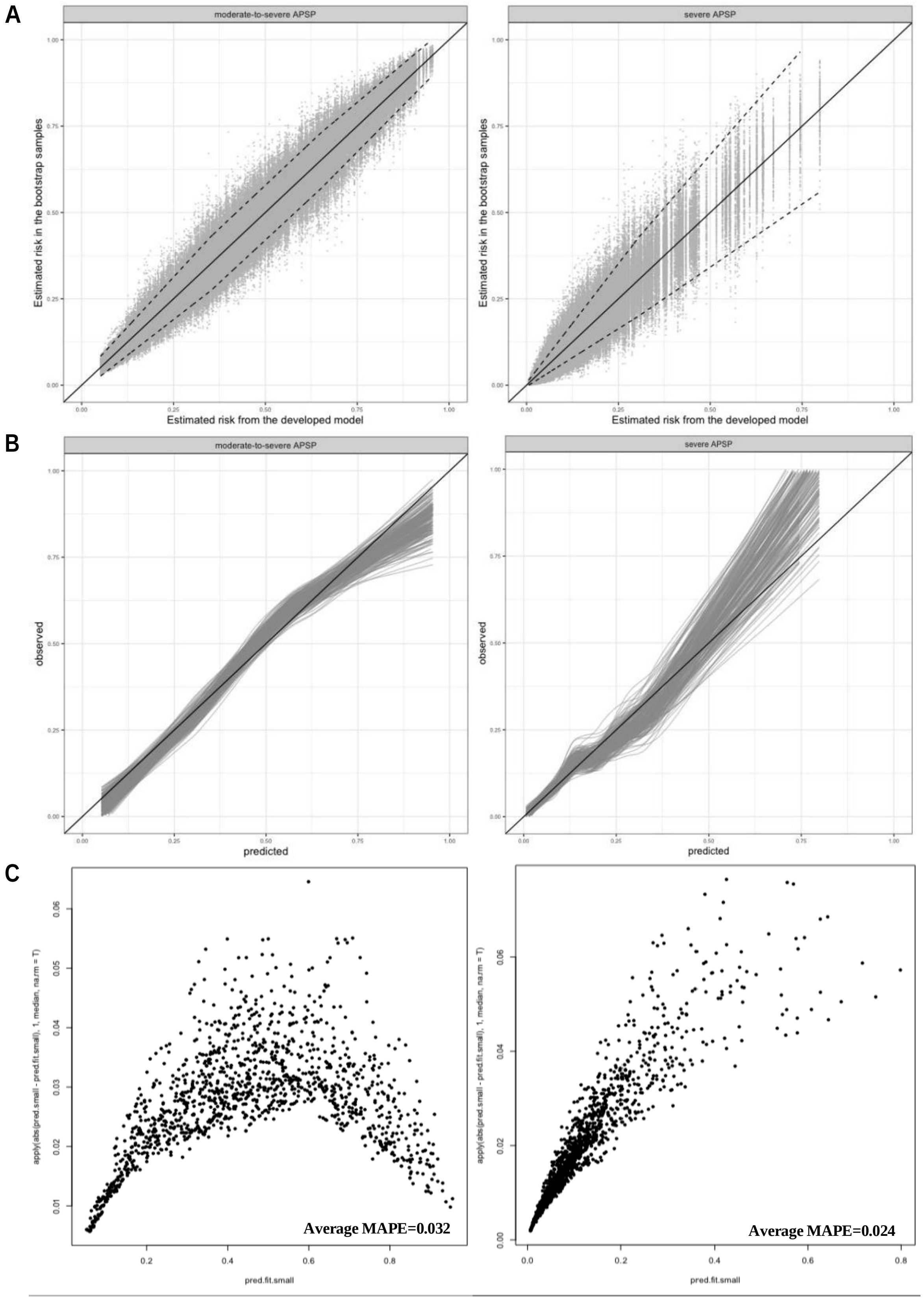

Figure S4. Instability plots for final APSP models. Model-building steps were repeated in each of 200 bootstrap samples to produce multiple bootstrap models and derive instability assessment plots. A) Individual prediction instability plots for moderate-to-severe (left) and severe (right) APSP models; B) Calibration instability plots for moderate-to-severe (left) and severe (right) APSP models; C) Mean absolute prediction error (MAPE) instability plots for moderate-to-severe (left) and severe (right) APSP models showing the average absolute difference in predictions across individuals in the developed models and the bootstrap models. The plots suggest predictions generated from the developed models are relatively stable and demonstrate reliability of individual risk estimates.

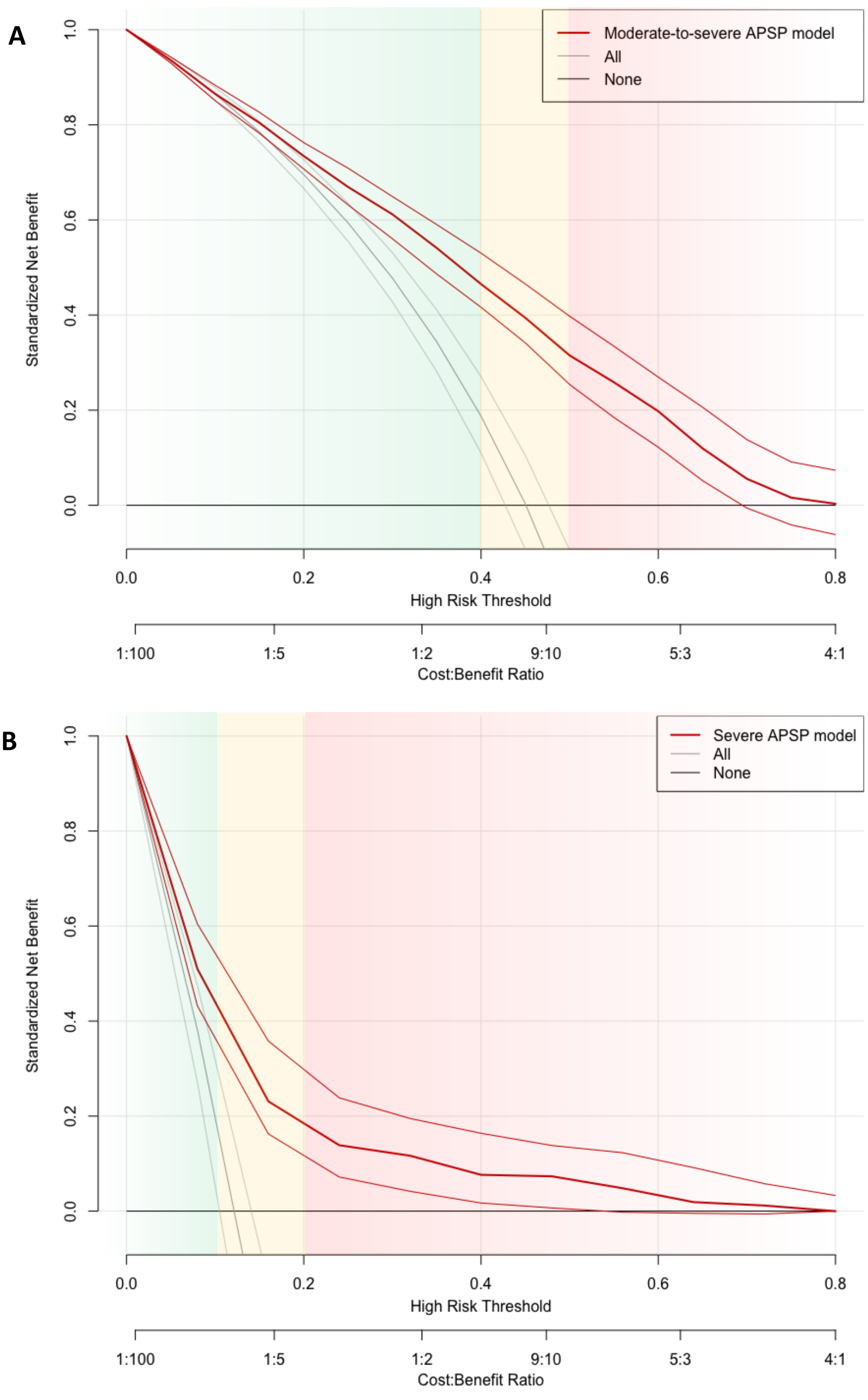

Figure S5. Decision-curve analysis. (A) Model predicting moderate-to-severe APSP; (B) Model predicting severe APSP, with 95% confidence intervals. The horizonal axis is the threshold estimated probability of APSP and the vertical axis is the net benefit NB. The plot compares the clinical benefit of three approaches: "treat all", "treat none" and decision based on the prediction model. The NB was standardized using the prevalence P of the outcome so that sNB has a maximum value of 1, known as relative utility.[37] For example, the model predicting moderate-to-severe APSP at a threshold probability of 40% where P = 45.1%, the sNB can be calculated as sNB = NB/P = 0.210/0.451 = 47%. This means, the prediction model offers the same sNB to the population as a policy that would result in the appropriate treatment for 47 cases while suggesting no inappropriate treatment (no false positives), per 100 patients. Suggested risk thresholds are highlighted (green = low, yellow = moderate, red = high).

Appendix B: Risk formulas and hypothetical patient examples

Risk formulas and hypothetical patient examples:

The following examples use hypothetical patients to demonstrate the intended clinical applicability of each model. Hypothetical patients 1 and 2 relate to moderate-to-severe APSP risk formula, hypothetical patients 3 and 4 relate to the severe APSP risk formula.

Age = Age in years, *CNB* = Central neuraxial block = 1, *EST* = Expected surgical technique (open = 1, minimally invasive = 0); *ESD* = Expected surgery duration; *NRS* = Average preoperative pain at rest in the past week (NRS 0–10); *Opioid* = Preoperative opioid in the past week = 1; *Other pain* = Other preoperative pain = 1; *PNB* = Peripheral nerve block = 1, *Sex* (female = 1, male = 0), *SG* = Surgery group (orthopedic = 1, non-orthopedic = 0)

Probability of moderate-to-severe APSP =
1/(1+exp(-{-0.597-0.012(**Age**)+0.375(**Sex**)+0.116(**NRS**)+0.585(**Opioid**)-0.675(**SG**)-0.325(**EST**)+0.021(**ESD**)+3.912e-06*pmax(**ESD**-30,0)^3-3.120e-05*pmax(**ESD**-60,0)^3+3.606e-06*pmax(**ESD**-90,0)^3-8.766e-07*pmax(**ESD**-170,0)^3-1.298(**CNB**)-0.678(**PNB**}))

Hypothetical patient 1: A 73-year-old male patient with average preoperative pain intensity in the surgical area at rest of NRS=2/10 and no preoperative opioid use in the past week, scheduled for an open orthopedic procedure with an expected duration of 50 minutes and is expected to receive a neuraxial block, has a predicted risk of 8% for moderate-to-severe APSP (defined as NRS ≥4) within the initial 3 hours after surgery. This patient could be classified “low risk”.

Hypothetical patient 2: A 20-year-old female patient with average preoperative pain intensity in the surgical area at rest of NRS=4/10 and has reported use of opioid analgesics in the past week, is scheduled for a minimally invasive orthopedic procedure with an expected duration of 60 minutes without nerve block, has a predicted risk of 77% for moderate-to-severe APSP within the initial 3 hours after surgery. This patient could be classified “high risk”.

Probability of severe APSP =
1/(1+exp(-{-1.871-0.013(**Age**)-2.337e-06*pmax(**Age**-26,0)^3+6.491e-05*pmax(**Age**-58,0)^3-4.154e-06*pmax(**Age-**76,0)^3+0.387(**Sex**)+0.117(**NRS**)+0.489*(**Other pain**)+0.705(**Opioid**)-0.584(**SG**)-0.004(**ESD**)+4.131e-06*pmax(**ESD**-30,0)^3-9.364e-05*pmax(**ESD**-60,0)^3+5.645e-06*pmax(**ESD**-90,0)^3-4.128e-07*pmax(**ESD**-170,0)^3-0.918(**CNB**)-0.218(**PNB**)}))

Hypothetical patient 3: A 67-year-old male patient with no preoperative pain in the surgical area (NRS=0/10), no preoperative opioid use in the past week and no other pain, scheduled for laparoscopic lobectomy with an expected duration of 120 minutes and is expected to receive an intercostal nerve block, has a predicted risk of 8% for severe APSP (defined as NRS ≥7) within the initial 3 hours after surgery. This patient could be classified “low risk”.

Hypothetical patient 4: A 24-year-old female patient with average preoperative pain intensity in the surgical area at rest in the past week of NRS=3/10, no preoperative opioid use but reports other pain, is scheduled for a minimally invasive abdominal procedure with an expected duration of 120 minutes without nerve block, has a predicted risk of 43% for severe APSP within the initial 3 hours after surgery. This patient could be classified “high risk”.
